# Tumor size improves the accuracy of the prognostic prediction of T4a stage colon cancer

**DOI:** 10.1038/s41598-021-95828-4

**Published:** 2021-08-11

**Authors:** Yuexiang Liang, Qiang Li, Donglei He, Yong Chen, Jingquan Li

**Affiliations:** Department of Gastrointestinal Oncology Surgery, the First Affiliated Hospital of Hainan Medical University, Longhua Road, Longhua District, Haikou City, 570100 Hainan Province China

**Keywords:** Oncology, Cancer, Surgical oncology

## Abstract

The aim of this study was to evaluate the potential impact of tumor size on the long-term outcome of colon cancer (CC) patients after curative surgery. A total of 782 curatively resected T4a stage CC patients without distant metastasis were enrolled. Patients were categorized into 2 groups according to the best threshold of tumor size: larger group (LG) and smaller group (SG). Propensity score matching was used to adjust for the differences in baseline characteristics. The ideal cutoff point of tumor size was 5 cm. In the multivariate analysis for the whole study series, tumor size was an independent prognostic factor. Patients in the LG had significant lower 5-year overall survival (OS) and relapse-free survival (RFS) rates (OS: 63.5% versus 75.2%, *P* < 0.001; RFS: 59.5% versus 72.4%, *P* < 0.001) than those in the SG. After matching, patients in the LG still demonstrated significant lower 5-year OS and RFS rates than those in the SG. The modified tumor-size-node-metastasis (mTSNM) staging system including tumor size was found to be more appropriate for predicting the OS and RFS of T4a stage CC than TNM stage, and the -2log likelihood of the mTSNM staging system was smaller than the value of TNM stage. In conclusion, tumor size was an independent prognostic factor for OS and RFS. We maintain that tumor size should be incorporated into the staging system to enhance the accuracy of the prognostic prediction of T4a stage CC patients.

## Introduction

Tumor size has been verified to be associated with survival in many types of malignancy, and it is regarded as “T” stage of many solid tumors including breast, lung and liver cancers in the tumor-node-metastasis (TNM) staging system of the Union for International Cancer Control (UICC)^1–5^. Despite the value of tumor size as a prognostic indicator in those solid tumors, the prognostic significance of tumor size in gastrointestinal tumors has not been widely realized. Deng et al*.*^6^ demonstrated that tumor size as a T stage could accurately predict the survival of gastric cancer patients and it was an independent prognostic factor. Kunisaki et al.^7^ found that tumor size was a reliable prognostic factor of gastric cancer, and thus suggested that it should be included in staging system. As for CC, few studies^8–16^ focused on tumor size. Saha et al*.*^8^ reported that tumor size positively correlated with grade, T stage and node stage, and it was inversely associated with survival.

As tumor size is usually associated with staging and other prognostic factors that were reported in previous studies^8–16^, it is crucial to adjust for the baseline feature imbalance between patients with larger tumor size and those with smaller one, especially in retrospective analysis. In the present study, we used both Cox proportional hazard regression analysis and propensity score method to overcome bias due to different distribution of covariates for the groups. The purpose of this study was to evaluate the potential impact of tumor size on the long-term outcome of CC patients after curative surgery in a single center in China.

## Material and methods

### Patients

**Figure 1:**
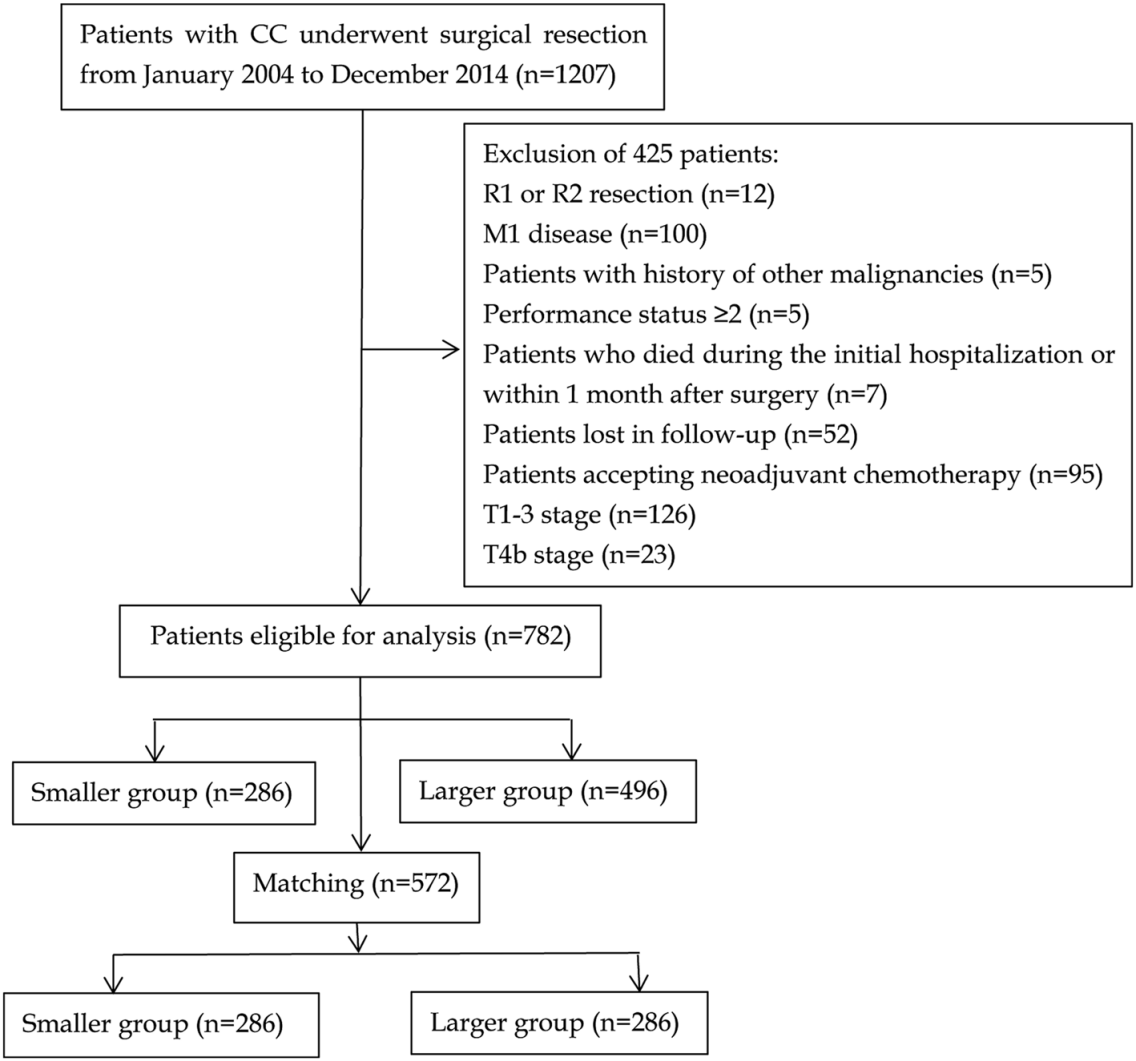
The criteria for inclusion and exclusion of all patients.

This study was reviewed and approved by the Ethics Committee of the First Affiliated Hospital of Hainan Medical University. All the patients signed an informed consent form for the operation including surgical procedure. All processes involved in this study were in accordance with the standards of the institutional Ethics Committee. A total of *1207* patients with CC who underwent surgical resection at the First Affiliated Hospital of Hainan Medical University between January 2004 and December 2014 were eligible for this study. The flow chart and exclusion criteria of this study were shown in Fig. 1. After exclusion of 425 patients, ultimately, a total of 782 T4a stage CC patients were included in this study.

### Evaluation of clinicopathological variables and survival

Clinicopathological features studied included the following 13 factors: sex, age at surgery, tumor location, tumor size, histology, lymphovascular invasion, lymph node metastasis, number of lymph nodes retrieval, surgical procedure, postoperative complications, postoperative adjuvant chemotherapy, preoperative serum carbohydrate antigen 19-9 (CA19-9) level and carcinoembryonic antigen (CEA) level.

All the patients underwent curative colonectomy plus complete mesocolic excision and lymph node dissection. The tumors were staged according to the eighth edition of the UICC TNM classification system. Tumors were classified into two groups based on WHO two-tier classification of histology grade: low grade and high grade. The indications of postoperative adjuvant chemotherapy included stage III patients and stage II patients with high risk factors for recurrence, such as poor histological differentiation, lymphovascular invasion, perineural invasion, preoperative bowel obstruction, and less than 12 lymph nodes examined, etc. However, whether the patients eventually received postoperative adjuvant chemotherapy was based on the patient's willingness, age, comorbid underlying diseases, physical status and pathological stages.

### Measurement of tumor size

The resected specimen was opened along the longitudinal axis of the colon wall on the opposite side of the tumor. Then, the opened colon was placed on a flat board with the mucosal side facing up. During the examination from the mucosal side, the longest tumor diameter was measured and regarded as tumor size in this study.

### Follow up

The patients were followed up by the attending physician and the research nurse of our department. To increase the follow-up rate, methods such as telephone, message, correspondence and outpatient department visits were used together. The patients were followed up every 3 months up to 2 years after surgery, then every 6 months up to 5 years, and then every year or until death. Physical examination, laboratory test (including assessing CEA and CA19-9), abdominal ultrasound, chest and abdominal computed tomography (CT) were performed at each visit, while endoscopy was obtained every year. The OS rate was calculated from the day of surgery until time of death or final follow-up. The date of final follow-up was December 31, 2019.

### Statistical analysis

For continuous variables, which were presented as mean ± standard deviation (SD), parametric analysis was performed using Student’s t test. Categorical variables were analyzed by means of the chi-square or Fisher’s exact test. Survival curves were calculated using the Kaplan–Meier method based on the length of time between primary surgical treatment and final follow-up, recurrence or death. The log-rank test was used to assess statistical differences between curves. Independent prognostic factors were identified by the Cox proportional hazard regression model. To overcome bias due to the different distribution of covariates for the two groups, the propensity score analysis was used to obtain a one-to-one match by using the nearest-neighbor matching method. And we imposed a caliper of 0.25 of the sd of the logit of the propensity score. Variables involved in the propensity model were sex, age at surgery, tumor location, tumor size, histology, lymphovascular invasion, lymph node metastasis, number of lymph nodes retrieval, surgical procedure, postoperative complications, postoperative adjuvant chemotherapy, preoperative serum CA19-9 level and CEA level. To compare our suggested new modified tumor-size-node-metastasis (mTSNM) staging system with the eighth edition of TNM stage, the -2 log likelihood, hazard ratio (HR) value, and 95% confidence interval (CI) related to the Cox regression model were used for measuring homogeneity and discriminatory ability. Smaller values of -2log likelihood indicated a better model for predicting outcome. *P* < 0.050 (bilateral) was considered statistically significant. The statistical analysis was performed using the statistical analysis program package SPSS 22.0 (SPSS, Chicago, IL).

## Results

### Clinicopathological features and survival of the whole study series

The median follow-up was 67 (range: 5–105) months. The 5-year OS and RFS rates were 67.8% and 64.6%, respectively. Of the 782 patients, 439 were male (56.1%), and 343 were female (43.9%). The age ranges from 26 to 83 years old, with a median age of 61 years. Of the 782 patients with curative resection, 597 patients had laparoscopic surgery, and 185 patients underwent open surgery. Among them, 601 patients accepted postoperative adjuvant chemotherapy with FOLFOX6, XELOX or capecitabine.

The mean ± SD tumor size was 6.17 ± 2.59 cm (range 0.80–17.00 cm). To identify the optimal cutoff points for tumor size, the cut-point survival analysis was adopted, and survival rates were calculated at each 1 cm interval. The tumor size with the highest χ^2^ value was regarded as the optimal threshold of classification. After numerous evaluations, the optimal thresholds were determined by the best cutoff approach in terms of the log-rank test. The ideal tumor size cutoff value was 5 cm in this study. The tumor size intervals were S1, < 5 cm and S2, ≥ 5 cm. All of the patients were categorized based on their tumor size intervals into one of the two groups: the larger group (LG) or the smaller group (SG). Clinicopathologic variables were compared in the left columns of Table 1. There was no statistical difference in sex, age at surgery, lymph node retrieval (≥ 12 *vs* < 12), lymph node metastasis, preoperative serum CEA level and postoperative adjuvant chemotherapy between the two groups. Compared with the SG, the number of lymph node retrieval in LG was larger (16.7 ± 7.5 *vs* 15.1 ± 5.6, *P* = 0.001), the ratio of tumor located at right colon was higher (65.3% *vs* 54.5%, *P* = 0.003), but the percentage of laparoscopic surgery was smaller (71.4% *vs* 85.0%, *P* < 0.001). Besides, high grade of histology (39.1% *vs* 29.4%, *P* = 0.006), lymphovascular invasion (18.1% *vs* 11.2%, *P* = 0.010), elevated CA19-9 (21.8% *vs* 13.3%, *P* = 0.003) and postoperative complications (10.3% *vs* 2.8%, *P* < 0.001) were more prevalent in larger tumors.

**Table 1:** *C*linicopathological features of T4a CC patients grouped by tumor size: data are reported for the whole study series and for one-to-one propensity-score matched pairs.

Characteristics	Whole study series			Matched pairs (Case–control Method)		
	SG(n = 286)	LG(n = 496)	P	SG(n = 286)	LG(n = 286)	P
Sex			0.118			0.397
Male/female	171/115	268/228		171/115	161/125	
Age at surgery (yr)			0.648			0.485
≥ 65/< 65	98/188	178/318		98/188	106/180	
Mean age at surgery			0.087			0.994
Mean ± SD	60.8 ± 11.1	59.3 ± 12.7		60.8 ± 11.1	60.8 ± 11.4	
Tumor location			0.003			0.448
Right colon/left colon	156/130	324/172		156/130	165/121	
Histology			0.006			0.260
Low grade/high grade	202/84	302/194		202/84	214/72	
Lymphovascular invasion			0.010			0.605
Present/absent	32/254	90/406		32/254	36/250	
Number of lymph nodes retrieval			0.001			0.144
Mean ± SD	15.1 ± 5.6	16.7 ± 7.5		15.1 ± 5.6	15.9 ± 7.2	
lymph nodes retrieval			0.472			0.288
> 12/≤ 12	209/77	374/122		209/77	220/66	
Lymph node metastasis			0.167			0.520
N0/N1/N2	193/67/26	354/89/53		193/67/26	198/57/31	
CEA level			0.787			0.556
Elevated(≥ 5.0 ng/ml) / Normal(< 5.0 ng/ml)	124/162	220/276		124/162	131/155	
CA19-9 level			0.003			0.406
Elevated(≥ 37.0 U/ml)/Normal(< 37.0 U/ml)	38/248	108/388		38/248	45/241	
Surgical procedure			< 0.001			0.064
Open/laparoscopic	43/243	142/354		65/221	60/226	
Postoperative chemotherapy	74.1	78.4	0.170			0.138
Yes/no	212/74	389/107		212/74	227/59	
Postoperative complications			< 0.001			0.136
Present/absent	8/278	51/445		8/278	15/271	

**Figure 2:**
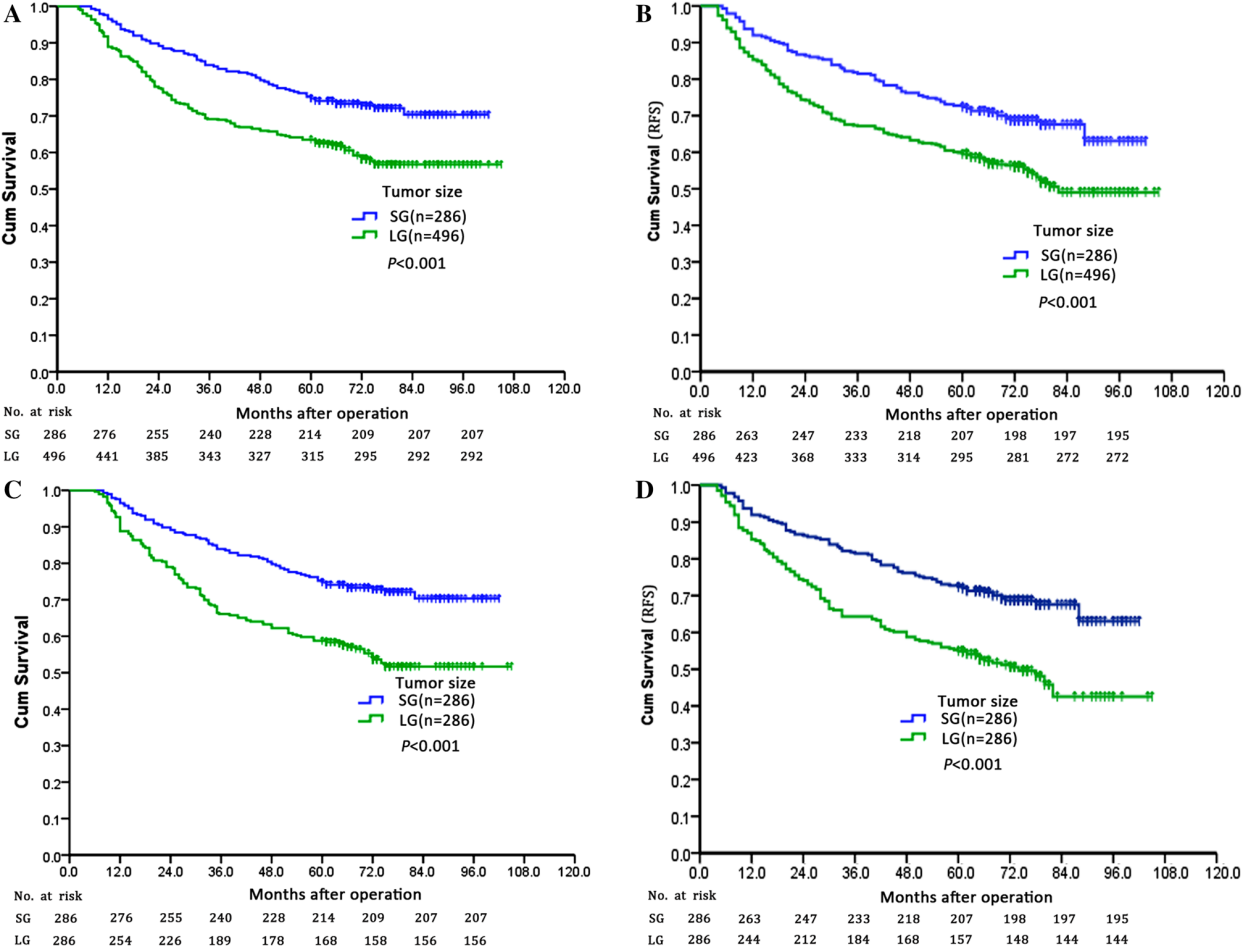
Prognosis of CC patients who underwent curative surgery. Patients were categorized into two groups according to the tumor size: SG and LG. (**A**) Overall survival curve for all patients: the 5-year OS rates were 75.2% and 63.5% for SG and LG, respectively (*P* < 0.001). (**B**) Relapse-free survival for all patients: the 5-year RFS rates were 72.4% and 59.5% for SG and LG, respectively (*P* < 0.001). (**C**) Overall survival curve for matched patients: the 5-year OS rates were 75.2% and 58.7% for SG and LG, respectively (*P* < 0.001). (**D**) Relapse-free survival for matched patients: the 5-year RFS rates were 72.4% and 54.9% for SG and LG, respectively (*P* < 0.001).

**Figure 3:**
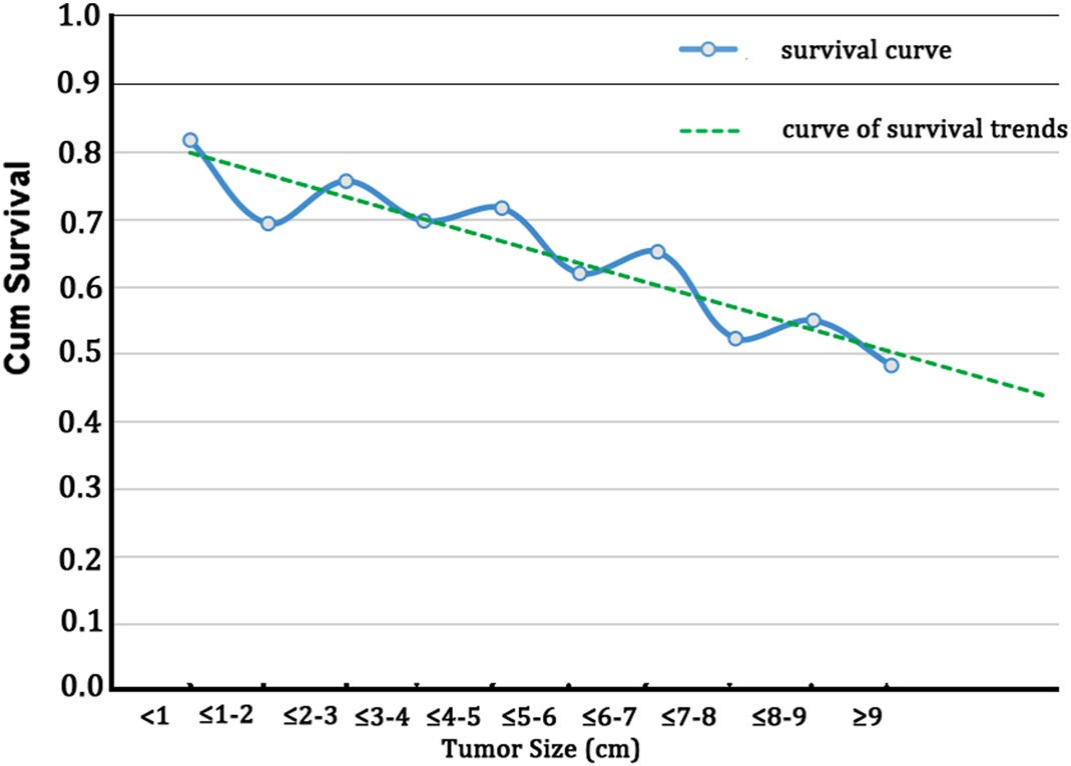
Distribution of cumulative survival by 1 cm tumor size intervals.

In the whole study population, the 5-year OS and RFS rates of LG were significantly lower than that of SG (OS: 63.5% versus 75.2%, *P* < 0.001; RFS: 59.5% versus 72.4%, *P* < 0.001, Fig. 2A-B). In the univariate analysis, the following 12 factors had a significant impact on OS and RFS: age at surgery (< 65 *versus* ≥ 65), tumor location, tumor size (< 5 *versus* ≥ 5 cm), histology, N stage, lymphovascular invasion, lymph node retrieval (> 12 *versus* ≤ 12), surgical procedure, CEA level, CA19-9 level, postoperative complications and postoperative adjuvant chemotherapy (Table 2). Multivariate analysis confirmed that tumor size was an independent prognostic factor for OS (HR was 1.433 for LG, *P* = 0.014) and RFS (HR was 1.448 for LG, *P* = 0.007), as were the following: N stage, lymphovascular invasion, lymph node retrieval, CA19-9 level, postoperative complications, postoperative adjuvant chemotherapy. The correlation between the 5-year OS rate and tumor size according to 1 cm intervals was analyzed. All of the patients were divided into ten groups according the 1 cm tumor size intervals. As shown in Fig. 3, the 5-year OS rate tended to decrease as tumor size increased (Fig. 3).

**Table 2:** Univariate and multivariate survival analysis in the whole study series.

Characteristics	n (%)	OS					RFS				
		5-year OS (%)	Univariate analysis		Multivariate analysis		5-year RFS (%)	Univariate analysis		Multivariate analysis	
			*HR(95%CI)*	*P*	*HR(95%CI)*			*HR(95%CI)*	*P*	*HR(95%CI)*	*P*
Sex											
Male	439(56.1)	67.9	1(ref)				64.7	1(ref)			
Female	343(43.9)	67.6	1.020(0.806–1.290)	0.871			64.1	1.007(0.806–1.259)	0.950		
Age at surgery											
< 65	506(64.7)	71.1	1(ref)		1(ref)		67.8	1(ref)		1(ref)	
≥ 65	276(35.3)	61.6	1.442(1.138–1.828)	0.002	1.358(1.048–1.759)	0.021	57.6	1.321(1.053–1.657)	0.016	1.234(0.964–1.578)	0.095
Primary tumor location											
Right colon	480(61.4)	63.1	1(ref)		1(ref)		59.8	1(ref)			
Left colon	302(38.6)	75.2	0.653(0.509–0.839)	0.001	0.910(0.689–1.201)	0.504	71.2	0.694(0.549–0.877)	0.002	0.945(0.729–1.225)	0.670
Tumor size											
SG (< 5 cm)	286(36.6)	75.2	1(ref)		1(ref)		72.4	1(ref)			
LG (≥ 5 cm)	496(63.4)	63.5	1.710(1.318–2.217)	< 0.001	1.433(1.076–1.908)	0.014	59.5	1.649(1.292–2.105)	< 0.001	1.448(1.107–1.894)	0.007
Histology											
Low grade	504(64.5)	72.4	1(ref)		1(ref)		68.3	1(ref)			
High grade	278(35.5)	59.4	1.589(1.256–2.010)	< 0.001	1.238(0.951–1.612)	0.112	56.8	1.496(1.196–1.871)	< 0.001	1.179(0.917–1.516)	0.199
N stage											
N0	547(69.9)	75.3	1(ref)		1(ref)		72.0	1(ref)			
N1	156(20.0)	59.0	1.835(1.386–2.429)	< 0.001	2.051(1.531–2.747)	< 0.001	54.5	1.819(1.396–2.369)	< 0.001	2.027(1537–2.672)	< 0.001
N2	79(10.1)	32.9	4.033(2.976–5.465)	< 0.001	4.369(3.192–5.979)	< 0.001	29.1	3.945(2.941–5.290)	< 0.001	4.360(3.222–5.901)	< 0.001
Lymphovascular invasion											
Absent	660(84.4)	71.1	1(ref)		1(ref)		67.4	1(ref)			
Present	122(15.6)	50.0	2.209(1.542–2.670)	< 0.001	1.760(1.295–2.392)	< 0.001	46.7	1.843(1.409–2.412)	< 0.001	1.617(1.198–2.181)	0.002
lymph nodes retrieval											
≤ 12	199(25.4)	58.8	1(ref)		1(ref)		54.8	1(ref)			
> 12	583(74.6)	70.8	0.674(0.524–0.866)	0.002	0.579(0.448–0.749)	< 0.001	67.4	0.719(0.565–0.914)	0.007	0.625(0.489–0.800)	< 0.001
CEA level											
Normal(< 5.0 ng/ml)	438(56.0)	72.8	1(ref)		1(ref)		70.1	1(ref)			
Elevated(≥ 5.0 ng/ml)	344(44.0)	61.3	1.462(1.158–2.847)	0.001	1.141(0.876–1.486)	0.329	57.3	1.422(1.140–1.774)	0.002	1.176(0.916–1.508)	0.203
CA19-9 level											
Normal(< 37.0U/ml)	636(81.3)	70.8	1(ref)		1(ref)		67.5	1(ref)			
Elevated(≥ 37.0U/ml)	136(18.7)	54.8	1.703(1.299–2.232)	< 0.001	1.514(1.130–2.027)	0.005	50.0	1.670(1.289–2.162)	< 0.001	1.485(1.123–1.964)	0.006
Surgical procedure											
laparoscopic	597(76.3)	69.3	1(ref)		1(ref)		66.3	1(ref)			
Open	185(23.7)	62.7	1.356(1.044–1.760)	0.022	1.223(0.934–1.602)	0.144	58.4	1.310(1.021–1.680)	0.034	1.151(0.890–1.4888)	0.284
Postoperative complications											
No	723(92.5)	70.3	1(ref)		1(ref)		66.4	1(ref)			
Yes	59(7.5)	37.3	2.385(1.687–3.373)	< 0.001	1.815(1.247–2.642)	0.002	37.3	2.141(1.518–3.019)	< 0.001	1.713(1.184–2.479)	0.004
Postoperative adjuvant chemotherapy											
No	181(23.1)	59.1	1(ref)		1(ref)		54.7	1(ref)			
Yes	601(76.9)	70.4	0.639(0.494–0.826)	0.001	0.667(0.505–0.880)	0.004	67.1	0.633(0.496–0.808)	< 0.001	0.632(0.487–0.821)	0.001

### Characteristics and survival of matched pairs

**Figure 4:**
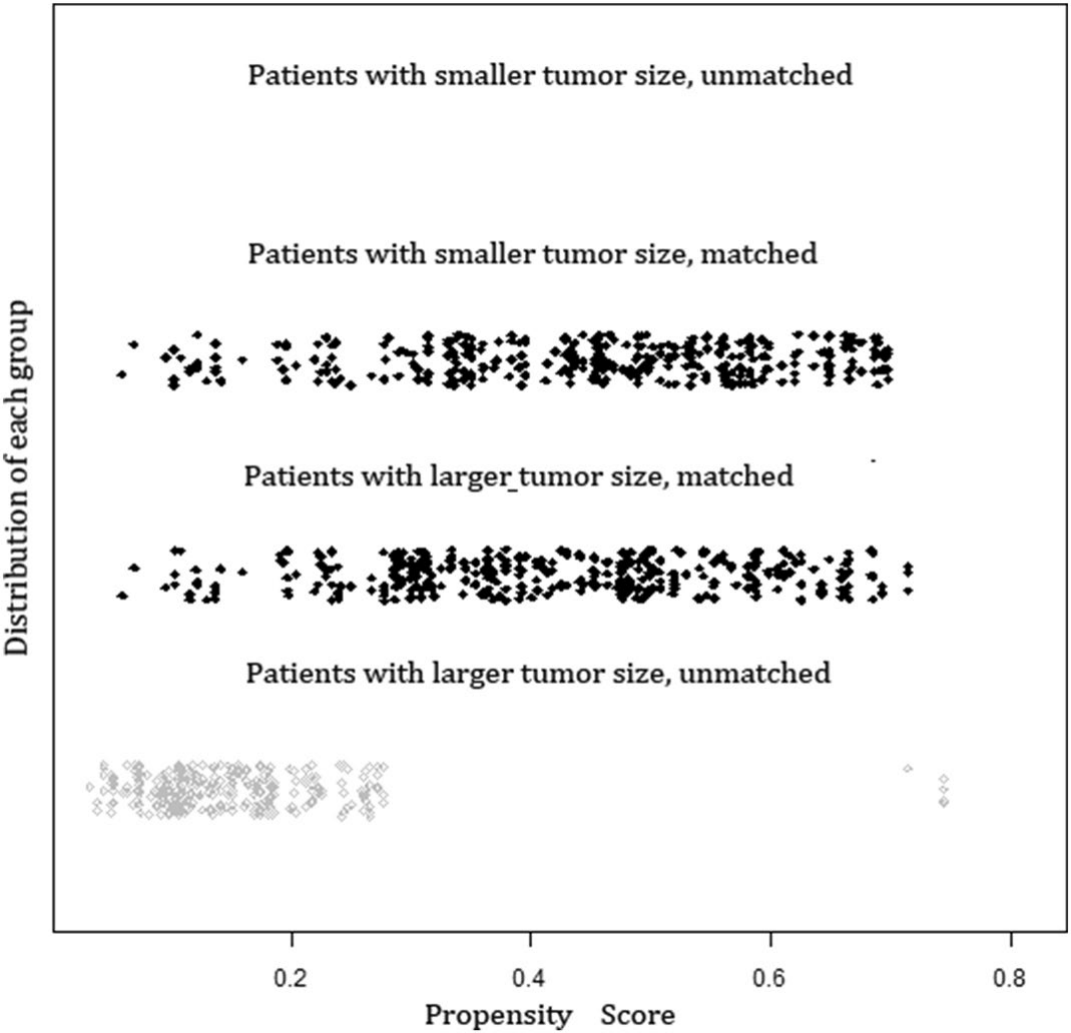
Distribution of the propensity scores. Each circle represents one patients.

We selected 286 patients for the LG for one-to-one matching with the SG by using propensity scores. The median follow-up was 66 (range: 6–105) months. Patients characteristics after matching were shown in the right column of Table 1. Of the 496 patients in the LG group, 286 cases were matched with 286 patients of the SG after adjustment of covariates. The adjusted propensity score of the LG was approximately identical to that of the SG (0.425 ± 0.103 *versus* 0.425 ± 0.098, *P* = 0.927). Figure 4 displayed the distribution of the propensity scores in the matched and unmatched patients in the LG and those in the SG. All covariates were evenly distributed in the two matching groups. Following factors of matched patients in LG were similar to that of SG: sex, mean age, tumor location, histological type, lymphovascular invasion, number of lymph nodes retrieval, lymph node metastasis, CEA level, CA19-9 level, surgical procedure, postoperative complications and postoperative adjuvant chemotherapy.

After matching, patients of LG still demonstrated significantly lower 5-year OS and RFS rates than those of SG (OS: 58.7% versus 75.2%, *P* < 0.001; RFS: 54.9% versus 72.4%, *P* < 0.001, Fig. 2C-D).

### Incorporation of the tumor size of CC into the eighth edition UICC TNM staging system

**Figure 5:**
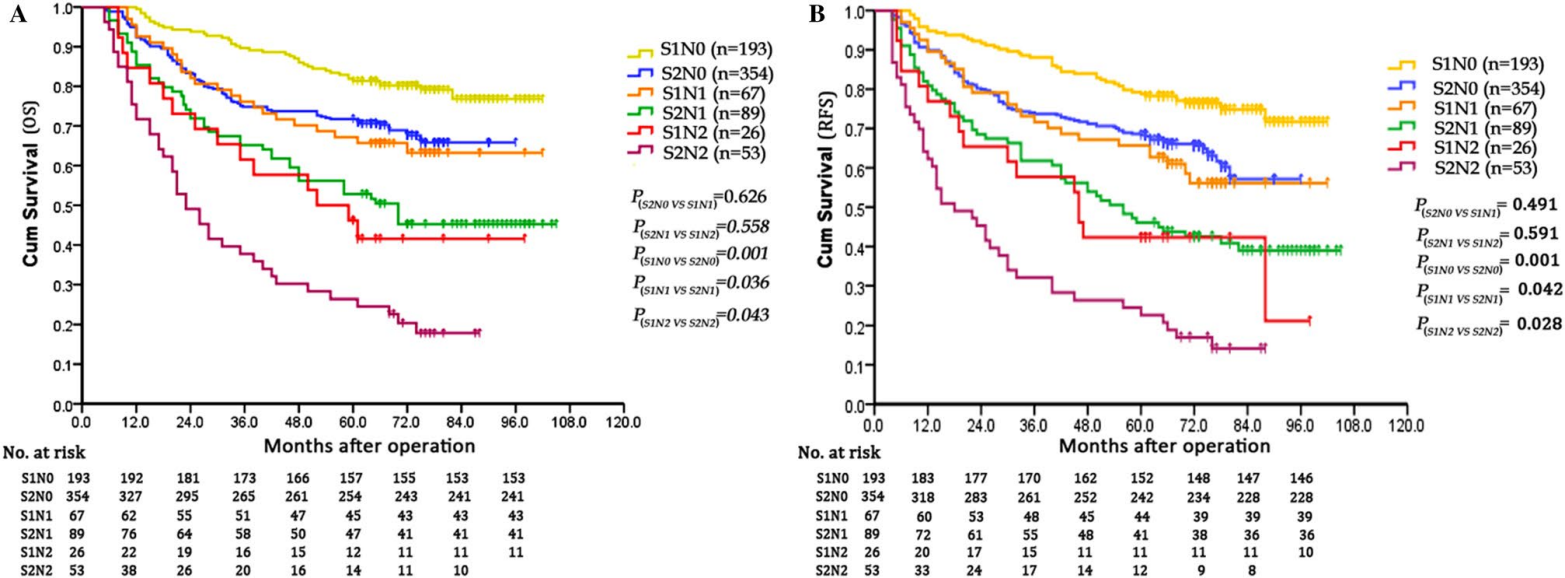
Comparison of survival curves of T4aM0 stage patients with different N stages and tumor sizes. (**A**) The OS of N0-stage patients with S2-size was similar to that of N1-stage patients with S1-size (*P* = 0.626). The OS of N1-stage patients with S2-size was equal to that of N2-stage patients with S2-size (*P* = 0.558). (**B**) The RFS of N0-stage patients with S2-size was similar to that of N1-stage patients with S1-size (*P* = 0.491). The RFS of N1-stage patients with S2-size was equal to that of N2-stage patients with S2-size (*P* = 0.591).

The OS and RFS of N0-stage patients in LG were similar to that of N1-stage patients in SG. The OS and RFS of N1-stage patients in LG were equal to that of N2-stage patients in SG (Fig. 5). According to the results, we incorporated tumor size into the eighth edition of UICC TNM stage and introduced the newly modified tumor-size-node-metastasis (mTSNM) stage. The new mTSNM staging system was presented in Table 3. For T4a stage CC patients without distant metastasis, the mTSNM stages were defined as follows: mIIB, N0-stage patients with tumor size < 5 cm; mIIIA, N0-stage patients with tumor size ≥ 5 cm and N1-stage patients with tumor size < 5 cm; mIIIB, N1-stage patients with tumor size ≥ 5 cm and N2-stage patients with tumor size < 5 cm; and mIIIC, N2-stage patients with tumor size ≥ 5 cm.

**Table 3:** T4a CC patients were divided into two groups according to the tumor size: S1 and S2 groups. S was included into staging system, then the new stages were suggested.

S	N0	N1	N2
S1	IIB	IIIA	IIIB
S2	IIIA	IIIB	IIIC

S: tumor size.

**Figure 6:**
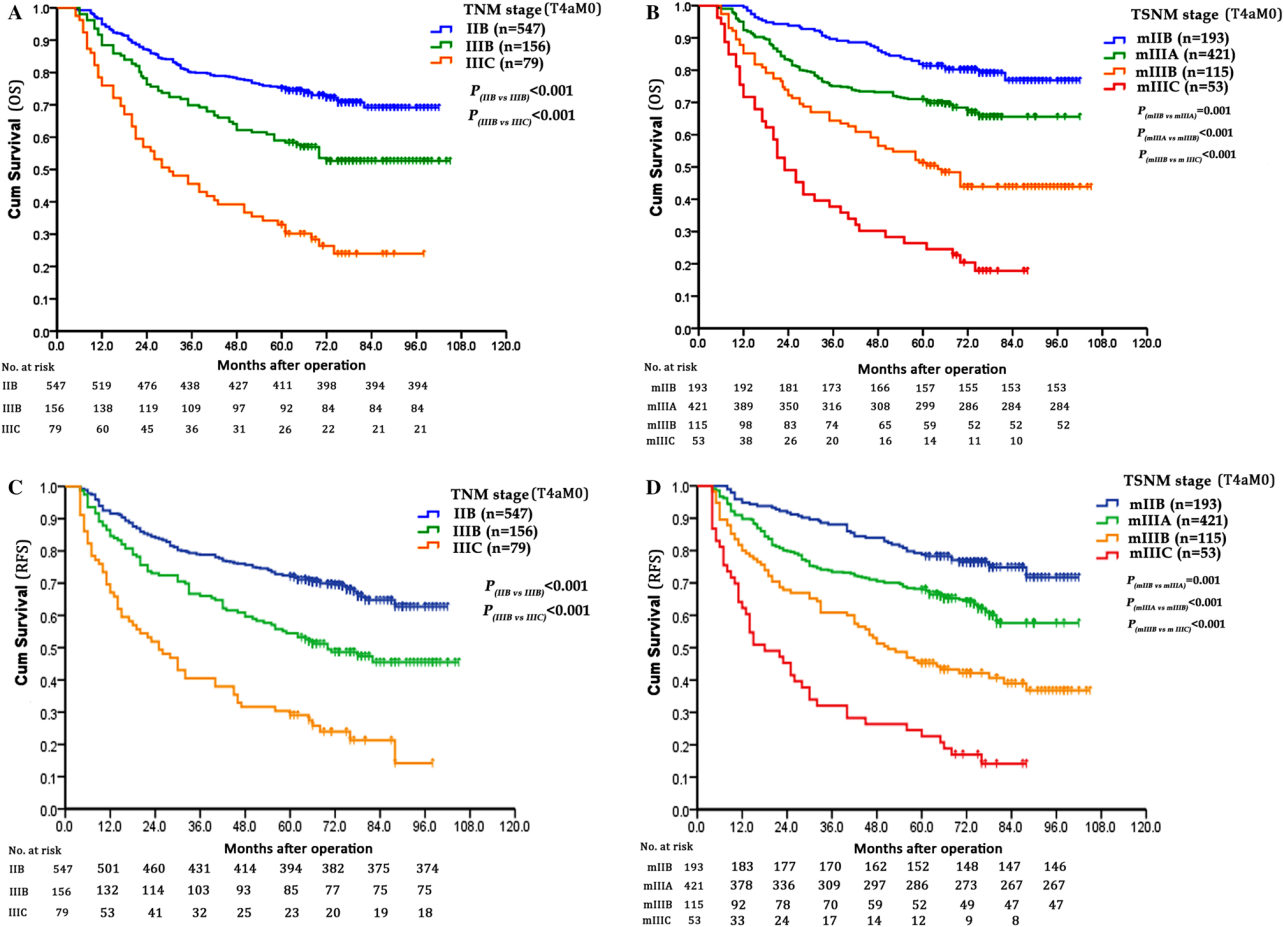
Survival curves of patients according to different tumor stages. (**A**) OS curves of TNM stage (*P* < 0.001). (**B**) OS curves of mTSNM stage (*P* < 0.001). (**C**) RFS curves of TNM stage (*P* < 0.001). (**D**) RFS curves of mTSNM stage (*P* < 0.001).

**Figure 7:**
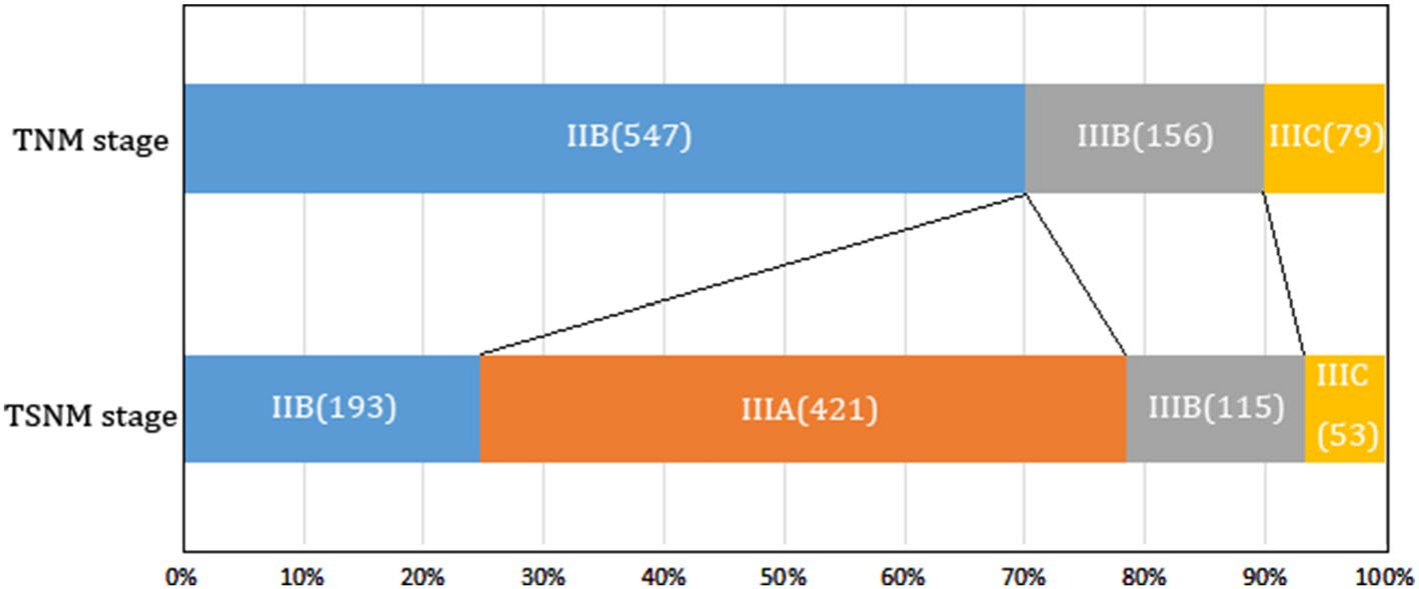
Patients distribution of different classification system. In the TNM staging system, patients were staged from IIB to IIIC, and the largest subgroup was IIB stage, however, there was no IIIA stage. In the mTSNM staging system, patients were continuously distributed from IIB to IIIC stage and the largest subgroup was IIIA stage.

The prognostic values of the TNM stage and mTSNM stage were evaluated by univariate and multivariate analyses. In the TNM stages, the 5-year OS rates were 73.5%, 59.0% and 32.9% in the IIB, IIIB and IIIC stages, respectively (χ^2^ = 95.542, *P* < 0.001), and the 5-year RFS rates were 72.8%, 54.5% and 30.4% in the IIB, IIIB and IIIC stages, respectively (χ^2^ = 99.658, *P* < 0.001). In the mTSNM stages, the 5-year OS rates were 81.9%, 71.0%, 51.3% and 26.4% in the mIIB, mIIIA, mIIIB and mIIIC stages, respectively (χ^2^ = 120.375, *P* < 0.001), and the 5-year RFS rates were 79.3%, 68.2%, 45.2% and 24.5% in the mIIB, mIIIA, mIIIB and mIIIC stages, respectively (χ^2^ = 125.645, *P* < 0.001) (Table 4, Fig. 6A-D). As presented in Fig. 7, the largest subgroup in the TNM stage is IIB, wherase the largest subgroup in the mTSNM stage is IIIA. The differences in prognostic prediction between the TNM stage and the mTSNM stage were compared directly. The mTSNM stage was confirmed to be a more accurate prognostic classification for predicting the OS and RFS of T4a stage CC patients after curative resection than the TNM stage. The -2 log likelihood of the mTSNM stage was less than the value of the TNM stage (for OS: 3469.212 versus 3477.452; for RFS: 3919.911 versus 3942.910).

**Table 4:** Survival analysis of the 782 CC patients according to the TNM and mTSNM stages.

Staging system	Cases	OS						RFS					
		5-year OS (%)	Univariate analysis		Multivariate analysis			5-year RFS (%)	Univariate analysis		Multivariate analysis		
			χ^2^	P	HR(95% CI)	P	−2loglikelihood		χ^2^	P	HR(95% CI)	P	−2loglikelihood
TNM stage			95.542	< 0.001	1.512(1.374–1.663)	< 0.001	3477.452		99.658	< 0.001	1.498(1.367–1.641)	< 0.001	3942.910
IIB	547	75.3						72.8					
IIIB	156	59.0						54.5					
IIIC	79	32.9						30.4					
mTSNM stage			120.375	< 0.001	1.939(1.700–2.212)	< 0.001	3469.212		125.645	< 0.001	1.906(1.680–2.163)	< 0.001	3919.911
IIB	193	81.9						79.3					
IIIA	421	71.0						68.2					
IIIB	115	51.3						45.2					
IIIC	53	26.4						24.5

## Discussion

Tumor size, given as the maximum diameter of the tumor, was one of significant prognostic factors of many solid tumors^1–5^. As for gastrointestinal carcinoma, several studies^6–9^ affirmed that tumor size positively correlated with important prognostic factors and negatively impacted survival. However, comparing with depth of invasion, tumor size was not a better predictive factor, and its prognostic value was often neglected. Some researchers believed that tumor size was easily affected by many other factors including depth of invasion and lymph node metastasis, and it could not predict prognosis independently. In addition, it was difficult to reach consensus on the best cutoff points of tumor size worldwide^17,18^. At present, the prognostic value of tumor size in CC remained controversial. Usually prognosis of CC patients without serosa invasion was rarely affected by tumor size. Patients with T4b disease tended to receive preoperative neoadjuvant chemotherapy, which was affirmed to be safe and associated with potential survival benefit, and had a significant impact on tumor size^19–21^. Therefore, we particularly focused on T4a stage CC patients in this study. We found that baseline characteristics were extremely imbalanced between LG and SG. To eliminate bias due to the different distribution of covariates between the two groups, propensity score matching and multivariate Cox regression analysis were applied together. It was confirmed that tumor size was one of independent prognostic factors for CC. Incorporation of tumor size into the eighth edition of TNM stage could improve the accuracy of the prognostic prediction of T4a stage CC patients.

Tumor size could be objectively and easily measured. Its prognostic value and clinical significance had been widely evaluated in gastric cancer. Several studies^22–24^ confirmed that larger tumor size was associated with significantly poorer OS than smaller tumor size in a given subset of gastric cancer, such as Borrmann type III, node-negative, or T4aN0M0 stage disease. As digestive tract tumors, CC and gastric cancer have some similar clinicopathological features. Besides, tumor size is closely related to surgical methods and scope of CC. The prognostic value of tumor size in CC should be explored as well. Theoretically, tumor size increases with tumor progression, and patients with larger tumor size usually have a poorer prognosis than those with smaller one. Previous studies^6–9^ had confirmed this theory and concluded that poorer prognosis of larger tumor was associated with tumor necrosis, iron deficiency anemia, tumor grade and a more aggressive underlying biology. In contrast, some studies^10–12,15,16^ reveled that patients with smaller tumor size had a worse prognosis in a given subset of CC, such as positive lymph node and IIA stage disease. In these studies^10,13–15^, the researchers considered that the poor prognosis of CC patients with smaller tumor size in the same stage was related to a more biologically aggressive phenotype. In addition, surgeons were more likely to treat larger tumors more aggressively by extending lymph node dissection or postoperative adjuvant chemotherapy, which might account for the better survival of patients with larger tumors^15^. As tumor size was associated with lymph node metastasis, depth of invasion and other prognostic factors, it was crucial to balance the relevant factors in both groups. To the best of our knowledge, this study was the first analysis using propensity score matching to assess the impact of tumor size on the prognosis of CC patients. We found that the incidence of lymphovascular infiltration, high grade of histology and postoperative complications were higher in patients with larger tumors. Besides, patients with larger tumors had significant lower 5-year OS and RFS rates than those with smaller tumors and tumor size was an independent prognostic factor of CC patients. This result was consistent with previous studies^8,9^. After matching, patients with larger tumor size still demonstrated significant lower 5-year OS and RFS rates than those with smaller tumor size (OS: 58.7% versus 75.2%, *P* < 0.001; RFS: 54.9% versus 72.4%, *P* < 0.001). Although previous studies had reported some possible causes of the prognostic impact of tumor size, so far, the mechanism remained unclear. Usually, the direction of primary tumor infiltration includes along the intestinal wall and perpendicular to the intestinal wall. The former forms tumor size, while the latter contributes to depth of invasion. For tumors at the same T stage, the prognosis of patients with larger tumor size is worse than that of patients with smaller tumor size, which may be due to the larger tumor burden and higher possibility of invading vascular and lymphatic channels in larger tumors. In addition, larger tumors were associated with significant reductions in serum hemoglobin and albumin, but increased chances of bowel obstruction. These factors had been affirmed to be associated with poor prognosis of CC in previous studies^25–28^. We believe that the depth of tumor invasion plays a major role in the prognosis of CC, but the effect of tumor size on survival can not be ignored.

Tumor size, as a T stage of many solid tumors, had been incorporated into the TNM staging system. As for digestive tract tumors, the depth of invasion plays a greater role in prognosis than the tumor size, and it is regarded as T stage. The role of tumor size is often ignored. However, several studies^6,29^ confirmed that incorporation of tumor size into the staging system could improve the prognostic prediction of gastric cancer. Deng et al*.*^6^ even used tumor size as a T classification and established a new tumor size-node-metastasis classification system. They found that the new tumor size-node-metastasis classification could accurately evaluate prognosis and provide very powerful discrimination of patients’ OS, as compared with TNM classification. Until now, few studies have incorporated tumor size into the staging system of colon cancer. In the present study, we found that the OS and RFS of N0-stage patients in LG were similar to that of N1-stage patients in SG, and the OS and RFS of N1-stage patients in LG were equal to that of N2-stage patients in SG. Based on the results, we incorporated tumor size into the TNM staging system and established a new mTSNM classification. It was affirmed that the mTSNM classification was a more appropriate prognostic classification to predict the OS and RFS of CC patients than the eighth edition of the TNM staging system. We believed that the current edition of the TNM staging system had following shortcomings. Firstly, it could not reflect the continuity of tumor progression. For example, the stage of T4aN0M0 patients was IIB, however, it crossed IIIA stage and jumped to IIIB and IIIC stages once lymph nodes were involved. Besides, the IIIA stage merely included T1N1-2aM0 and T2N1M0 patients, however, lymph node metastasis was rare in T1-2 stage patients. In our suggested mTSNM staging system, patients were continuously and uniformly distributed from IIB to IIIC stage and the largest subgroup was IIIA stage.

There were several limitations to our study. First and foremost was the limitations inherent to retrospective analyses. Moreover, as the sample size was relatively small, patients was simply divided into two groups based on best cutoff value of tumor size, more elaborate division of subgroups was not performed. Optimal cut-off values varied among different parts of the large bowel, usually decreasing from the right colon to the left, while tumor location was not concerned when identifying best threshold. Third, this study had a long period of inclusion and a higher fraction of T4a tumors than that reported in other studies, which could not be representative for the general population of CC patients. The economic level and medical conditions of Hainan were relatively poorer than other provinces in China. Routine physical examinations were rarely carried out, and most patients came to hospital when they had obvious symptoms. As a result, most patients were diagnosed at middle and late stages, which might account for the higher fraction of T4a tumors. Nevertheless, even with these limitations, our results suggested that tumor size was relevant in patients with CC.

## Conclusion

Tumor size is an independent prognostic factor and negatively impacts survival of CC patients. Prognostic impact of tumor size should be considered when making prognosis evaluation. Besides, we maintain that tumor size should be incorporated into the staging system to enhance the accuracy of the prognostic prediction of T4a stage CC patients. Further studies are still necessary to elucidate the mechanism of tumor size as a prognostic factor in CC.

## References

[1] Wei W (2018). Comparison of residual risk-based eligibility vs tumor size and nodal status for power estimates in adjuvant trials of breast cancer therapies. JAMA Oncol..

[2] Cortesi L (2013). Tumor size, node status, grading, HER2 and estrogen receptor status still retain a strong value in patients with operable breast cancer diagnosed in recent years. Int. J. Cancer..

[3] Pang Z (2017). Prognostic effects of preoperative obstructive pneumonitis or atelectasis and comparison with tumor size in non-small cell lung cancer. J. Thorac. Dis..

[4] Travis WD (2016). IASLC lung cancer staging project: proposals for coding T categories for subsolid nodules and assessment of tumor size in part-solid tumors in the forthcoming eighth edition of the TNM classification of lung cancer. J. Thorac. Oncol..

[5] Hwang S (2015). The impact of tumor size on long-term survival outcomes after resection of solitary hepatocellular carcinoma: single-institution experience with 2558 patients. J. Gastrointest. Surg..

[6] Deng J (2015). Tumor size as a recommendable variable for accuracy of the prognostic prediction of gastric cancer: a retrospective analysis of 1,521 patients. Ann. Surg. Oncol..

[7] Feng H (2020). Association of tumor size with prognosis in colon cancer: a surveillance, epidemiology, and end results (SEER) database analysis. Surgery.

[8] Saha S (2015). Tumor size predicts long-term survival in colon cancer: an analysis of the National Cancer Data Base. Am J Surg..

[9] Dai W (2020). The critical role of tumor size in predicting prognosis for T1 colon cancer. Oncologist.

[10] Lee SY (2018). Macroscopic serosal invasion and small tumor size as independent prognostic factors in stage IIA colon cancer. Int. J. Colorectal Dis..

[11] Wang Y (2015). Unfavorable effect of small tumor size on cause-specific survival in stage IIA colon cancer, a SEER-based study. Int. J. Colorectal Dis..

[12] Santullo F (2018). Tumor size as a prognostic factor in patients with stage IIa colon cancer. Am. J. Surg..

[13] Huang B (2016). Smaller tumor size is associated with poor survival in T4b colon cancer. World J. Gastroenterol..

[14] Yamanashi T (2018). Laparoscopic surgery for locally advanced T4 colon cancer: the long-term outcomes and prognostic factors. Surg. Today..

[15] Huang B (2016). Smaller tumor size is associated with poor survival in stage II colon cancer: an analysis of 7,719 patients in the SEER database. Int. J. Surg..

[16] Muralidhar V (2016). Association between very small tumor size and increased cancer-specific mortality in node-positive colon cancer. Dis. Colon Rectum..

[17] Kooby DA (2003). Biologic predictors of survival in node-negative gastric cancer. Ann. Surg..

[18] Huang KH (2009). Factors affecting recurrence in node-negative advanced gastric cancer. J. Gastroenterol. Hepatol..

[19] Silva R (2021). Does preoperative neoadjuvant chemotherapy impact short-term surgical outcomes in patients with locally advanced colon cancer?. Int. J. Colorectal Dis..

[20] Body A (2021). The role of neoadjuvant chemotherapy in locally advanced colon cancer. Cancer Manag Res..

[21] Cheong CK (2020). Neoadjuvant therapy in locally advanced colon cancer: a meta-analysis and systematic review. J. Gastrointest. Oncol..

[22] Hosoda K (2015). Preoperative tumor size is a critical prognostic factor for patients with Borrmann type III gastric cancer. Surg Today..

[23] Quan J (2013). The impact of tumor size on survival of patients with pT4aN0M0 gastric cancer. Am. Surg..

[24] Zhao LY (2016). A new predictive model combined of tumor size, lymph nodes count and lymphovascular invasion for survival prognosis in patients with lymph node-negative gastric cancer. Oncotarget.

[25] Chin CC (2010). Carcinoma obstruction of the proximal colon cancer and long-term prognosis–obstruction is a predictor of worse outcome in TNM stage II tumor. Int. J. Colorectal Dis..

[26] Li F (1999). Serum iron and ferritin levels in patients with colorectal cancer in relation to the size, site, and disease stage of cancer. J. Gastroenterol..

[27] Fujikawa H (2017). Prognostic impact of preoperative albumin-to-globulin ratio in patients with colon cancer undergoing surgery with curative intent. Anticancer Res..

[28] Boonpipattanapong T (2006). Preoperative carcinoembryonic antigen and albumin in predicting survival in patients with colon and rectal carcinomas. J. Clin. Gastroenterol..

[29] Liang Y (2019). Tumor size improves the accuracy of the prognostic prediction of lymph node-negative gastric cancer. J. Surg. Res..

